# Prevalence and factors associated with latent autoimmune diabetes in adults (LADA): a cross-sectional study

**DOI:** 10.1186/s12902-022-01089-1

**Published:** 2022-07-08

**Authors:** Anselmo M. Manisha, Aminiel R. Shangali, Sayoki G. Mfinanga, Erasto V. Mbugi

**Affiliations:** 1 Department Biochemistry, School of Medicine, Muhimbili University of Health and Allied Sciences, P.O. Box 65001, Dar es Salaam, Tanzania; 2 Department of Biochemistry and Physiology, Mwanza University, P.O. Box 1719, Mwanza, Tanzania; 3 Department of Parasitology, Microbiology and Immunology, Mwanza University, P.O. Box 1719, Mwanza, Tanzania; 4 National Institute for Medical Research, P.O. Box 3436, Dar es salaam, Tanzania; 5 Department of Epidemiology and Statistics, School of Public Health, Muhimbili University of Health, and Allied Science, P.O. Box 65001, Dar es Salaam, Tanzania

**Keywords:** LADA, GAD, Type 2 diabetes, Complication

## Abstract

**Background:**

The Latent Autoimmune Diabetes in Adults (LADA) is a slowly progressive Type 1 diabetes subgroup with onset during middle age. Studies report that about 10% of adults initially diagnosed with clinical Type 2 diabetes (T2D) have LADA. Inappropriate diagnosis and mismanagement of the LADA can increase the risk of diabetic complications, which affect the quality of life and is the cause of increased mortality. In low-income countries setting, data regarding the magnitude of LADA is limited. We carried out this study to estimate the burden of misdiagnosed LADA among T2D patients in selected health facilities in Dar es Salaam and to bring awareness to the use of Glutamic Acid Decarboxylase (GAD) autoantibody in screening for LADA.

**Methodology:**

We enrolled 186 phenotypically T2D patients in this cross-sectional study, through a standardized data collection tool we obtained participants’ demographic and clinical information. For testing GAD levels, we used a double-antibody Enzyme-Linked Immunosorbent Assay (ELISA). The Fisher’s Exact and student t-tests were used to test the significance of the statistical associations of the glycaemic control and diabetes complications between T2D and LADA.

**Results:**

Out of 186 patients, 156 gave conclusive GAD Ab ELISA reading with LADA accounting for 5.1% (95% CI: 2.5 - 10.0). The mean age of subjects was 54.3 years (Range: 33-85 years). The parameters such as mean age, family history of diabetes mellitus status, Fasting Blood Glucose, clinical characteristics, and complications did not show significant statistical differences between patients with LADA and Type 2 diabetes. However, all LADA- Human Immunodeficiency Virus (HIV) comorbid patients had retinopathy, which was statistically insignificant in 20 (87%) T2D-HIV comorbid patients (*p =* 0.669). Neither neuropathy, nephropathy, nor Diabetic Mellitus (D.M.) foot syndrome was observed among LADA-HIV comorbid patients. Nevertheless, 22 (95.7%), 3 (13%), and 2 (8.7%) of T2D-HIV comorbidity had neuropathy, nephropathy, or D.M. foot syndrome, respectively.

**Conclusions:**

The study established a LADA prevalence of 5.1% among T2D patients and has shown the role of GAD autoantibody in the screening for LADA. The study calls for a well- designed larger longitudinal study to generate strong evidence on the association of risk factors and complications associated with the LADA. This will develop robust evidence on the association of risk factors and complications associated with the LADA and T2D.

**Supplementary Information:**

The online version contains supplementary material available at 10.1186/s12902-022-01089-1.

## Background

The World Health Organization (WHO) step survey reports that inaccurate reporting of global diabetes prevalence to have significant impact on disease burden, monitoring, policy development and resource allocation [1]. This implies that regular surveys and possibly regional/country-based studies are critical in providing relevant information especially if appropriate survey methods are used. WHO has in addition, reported diabetes to be the ninth leading cause of death with an estimated 1.5 million diabetes-caused deaths [2]. The International Diabetics Federation (IDF) Atlas on the other hand, reports 10% of adults (20-79 years) living with diabetes in the world, of whom over 75% live in low- and middle-income countries claiming 6.7 million deaths in which 1 diabetic patient dies in every 5 seconds [3] In 2021 about 23.6 million people in Africa had diabetes with an estimate of projected increase to about 54.9 million by 2045 [4]. Change in lifestyle and urbanization in developing countries seems to be associated with the worsening burden of diabetes [5]. Reports in Tanzania show a prevalence of 9.1% of people living with diabetes and an increasingly steady rate of non-communicable diseases [6].

The WHO has classified LADA as a subgroup of Type 1 diabetes (T1D), sharing genetic similarities with both Type 1 and 2 diabetes mellitus (T2D) [7]. LADA patients have slow progressive β-Cell destruction, which eventually leads to insulin deficiencies and, therefore, the requirement for insulin therapy [8]. Furthermore, LADA is an adult based in its onset by which from the age of 30 years, the patients do not require treatment with insulin injection for the first 6 months after diagnosis. About 10% of adults initially diagnosed with T2D clinically have been defined as LADA patients [9–11].

The enzyme Glutamic acid decarboxylase (GAD) is found in neural and non-neural cells such as the pancreatic cells, oviduct, and testes [12]. The function of GAD in the islet β-Cells is not well documented, but its relevance may be in the regulation of paracrine effects in the secretion of insulin, glucagon, and somatostatin [13]. The GAD is among the major autoantigen in autoimmune diabetes and is considered the best sensitive autoantibody marker for LADA diagnosis [14, 15].

The LADA patients may present with clinical features like those found in T2D, but they require insulin treatment in prolonged management. There is limited evidence in developing countries, including Tanzania, concerning the outcome and complications of this subgroup of diabetes. Inappropriate diagnosis and mismanagement of the LADA can increase the risk of complications such as Coronary Heart Disease, Retinopathy, Nephropathy, Neuropathy, and Diabetics Mellitus (D.M.) foot which affect the management of quality of life, increase the cost of treatment, and may consequently result into death [11, 15–21].

Pending the absence of universally accepted criteria for the diagnosis of LADA, the study aimed to address the classification of the LADA, using antibodies to GAD [17] to exploit potential improvement in diagnosis, treatment choices, and management of diabetes. Furthermore, the study assessed the prevalence of LADA and its associated factors among patients with established Type-2 diabetes, which is reported in this manuscript.

## Methods

The study was a hospital-based cross-sectional study conducted in four diabetes clinics, namely, the Muhimbili National Hospital (MNH) and the three regional referral hospitals of Temeke, Amana, and Mwananyamala in Dar es Salaam, Tanzania. The city is located at 6°48′ S, 39°17′ E (− 6.8000, 39.2833) on a natural harbour on the coast of East Africa. The hospitals were selected based on the grounds that they serve as sites for screening, treatment, and management of diabetic patients.

The study aimed to determine the proportion of GAD positive among phenotypically Type 2 diabetic patients [22–25] to compare the glycaemic control of LADA to Type 2 diabetics’ patients, and to compare the complications of LADA to Type 2 diabetics’ patients with HIV and hypertension comorbidity conditions.

### Patients

The study enrolled 186 phenotypically T2D patients who provided consent conveniently sampled from all patients attending the diabetes clinics between February and June 2021. The ethical approval for the study was granted by the MUHAS Research Ethics Sub-Committee of the Senate Research and Publication Committee (Ethical Clearance Reference No.DA.282/298/01.C/). The inclusion criteria were diabetic patients with age onset of ≥30 years without ketoacidosis and non-insulin requiring for the first 6 months, disease duration not more than 5 years. Furthermore, the exclusion criteria were pregnancy, patients on steroids medication, cancer patients, patients with the stiff-person syndrome, cerebellar ataxia, epilepsy and neurological disorders unrelated to diabetes.

### Data collection

Participants were enrolled at MNH, Temeke, Amana, and Mwananyamala referral hospitals in a proportion of 3:3:2:3, respectively. The number of samples collected at each of the four clinics were dependent on the diabetic’s clinic days per week and the number of eligible patients attending the clinics per day.

A well-structured data collection tool compiled data from the questionnaire, medical records, and laboratory GAD Ab ELISA test results. Patients’ data, including age, sex, Body Mass Index (BMI), disease duration, and family history of D.M., were collected using questionnaires and cross-checked with hospital records to reduce recall bias. Other types of data such as glycaemic control, insulin treatment, duration of insulin therapy, history, and duration of hypertension were extracted from medical records and filled in the data collection tool. Histories of Diabetes Mellitus complications, including retinopathy, neuropathy, Diabetes Mellitus foot syndrome, and nephropathy, were also enquired/extracted from patients’ hospital records.

### Specimen collection

A phlebotomist collected 3mls of the participant’s blood sample in a plain vacutainer tube using the standard operating procedure set forth by the Tanzania ministry of health [26]. Sera from venous blood were extracted within 1 hour in the respective hospital laboratories. The serum was centrifuged for 3 minutes at 3000 rpm to remove the clot and was refrigerated at − 20 °C. The collected blood samples were only used for the purpose of the GAD autoantibody ELISA test in accordance with professional and ethical conduct.

### Laboratory procedures

Sera to be analysed were retrieved from storage at –20 °C refrigerator and left to thaw for 30 minutes at room temperature. About 10 μl of serum was sufficient for one assay. Test sera were brought to room temperature and mixed gently to ensure homogeneity when required. A double-antibody sandwich enzyme-linked immunosorbent one-step process assay (ELISA) was used to assay the level of glutamic acid decarboxylase 65(GAD65) in samples (Qayee-bio for life science). The reference range points for the double-antibody sandwich ELISA kit for Human GAD65 was 6.25 ng/ml – 200 ng/ml. The negatives in this study had titer values within the normal range which were defined as Type 2 Diabetes**,** and for positives had titer values above the normal range which were defined as LADA patients.

### Statistical analysis

The data were double entered in the Epi database, and post-entry validation was performed after cross-checking and correction to accuracy. Monitoring for the completeness of all questionnaires was done immediately after each interview or record was extracted from other hospital sources. Data was exported for further cleaning, coding, and analysis to a statistical and data sciences software (STATA for Windows version 14). The results were presented in percentages/proportions for the categorical variables. Descriptive statistics were used to examine the frequencies and associations between variables for quantitative data which were presented in mean ± standard deviation (S.D.) and median with inter quaternary range. The prevalence was calculated as the proportion of latent autoimmune diabetes in adults (LADA) among all-eligible phenotypic Type-2 diabetic patients reported with 95% CI around this points’ estimate. We performed cross-tabulation with stratification to assess the association for the risk factors, including clinical features and complications related to the LADA among diabetes Type 2 patients. The Fisher’s Exact test and students t-test were used to measure the association with risk factors. The sample statistics were considered statistically significant if *p* ≤ 0.05. The statistics were adjusted for interaction and potential cofounders for each respective factor and adjusted statistics to cater to potential confounders.

## Results

### Clinical histories and characteristics

Of 186 enrolled patients, females were 114 (61.3%), and males were 72 (38.7%). The mean age of the study participant was 54.3 years (Range: 33-85 years). The laboratory analysis gave a conclusive reading using a Thermo scientific ELISA reader of GAD in 156 patients, in which their LADA statuses were defined. The proportion of GAD positive (LADA) was 5.1% (95% CI: 2.5 - 10.0). Of the 8 LADA positive, 2 (25%) were males and 6 (75%) were females (*p =* 0.711).

All clinical histories and characteristics did not show significant statistical differences between patients with LADA and T2D. These clinical histories and characteristics included the mean age, age group distribution, history of smoking, alcohol, family history of diabetes mellitus, age of onset of diabetes, BMI, HIV, and T.B. status, BMI, acute symptoms before diagnosis, fasting blood sugar level and treatment choice. Other characteristics were diabetic complications such as retinopathy, neuropathy, nephropathy, Diabetes Mellitus (D.M.) foot, and hypertension (Table 1).

**Table 1:** The clinical histories and characteristics of the GAD-positive patients (LADA) in comparison with the GAD-negative patients (T2D) (*n* = 156)

Clinical histories and characteristics	Glutamate Decarboxylase status			
	**Total**	**LADA**	**TYPE 2 DM**	***p***-value
		***n*** **= 8**	***n*** **= 148**	
		**n (%)**	**n (%)**	
**Sex**				
Females	96 (61.5)	6 (75.0)	90 (60.8)	0.711
Males	60 (38.5)	2 (25.0)	58 (39.2)	
Age (mean ± SD)	54.3 ± 11.7	55.8 ± 16.6	54.2 ± 11.5	0.135^t^
BMI (mean ± SD)	27.5 ± 5.3)	28.5 ± 3.9	27.4 ± 5.4	0.309^t^
BMI distribution				
Underweight	7 (4.5)	0 (0)	7 (4.7)	1.00
Normal	45 (28.9)	2 (25.0)	43 (29.1)	
Overweight/ Obese	104 (66.7)	6 (75.0)	98 (66.2)	
Age onset of diabetes [median (IQR)]	52 (41- 62)	46.5 (38.3-68.5)	53 (41- 62)	0.698
Duration of diabetes in years [median (IQR)]	3 (1 – 4)	3.5(2.3-5)	3 (1 - 4)	0.803
History of alcohol	59 (37.8)	4 (50.0)	55 (37.2)	0.478
History of cigarettes	24 (15.4)	1 (12.5)	23 (15.5)	1.00
Family history with diabetes	72 (46.2)	3 (37.5)	69 (46.6)	0.726
Frequent urination	138 (88.5)	8 (100)	130 (87.8)	0.598
Ketonuria	16 (10.3)	2 (25.0)	14 (9.5)	0.192
Blurred vision	118 (75.6)	6 (75.0)	112 (75.7)	1.00
Increasing thirst	126 (80.8)	5 (62.5)	121 (81.8)	0.182
HIV positive (positive only)	24 (15.4)	1 (12.5)	23 (15.5)	1.00
MTB positive	12 (7.7)	0 (0)	12 (8.1)	1.00
Oral hypoglycaemia	139 (89.1)	8 (100)	131 (88.5)	0.60
Fasting blood sugar level at diagnosis (mean, SD)	21.8 (7.9)	23.3 (8.1)	21.8 (7.9)	0.948^t^

*LADA* Latent autoimmune diabetes in adults, *DM* Diabetes mellitus, *BMI* Body mass index, *SD* Standard deviation, *IQR* Inter quaternary range, *HIV* Human immunodeficiency virus, *MTB Mycobacterium tuberculosis*.

*Note: Superscript t indicates p-values obtained from the t-test, other P-values obtained from Fisher’s Exact Test*

### Comparison of characteristics between patients with LADA and type 2 diabetes mellitus

**Fig. 1:**
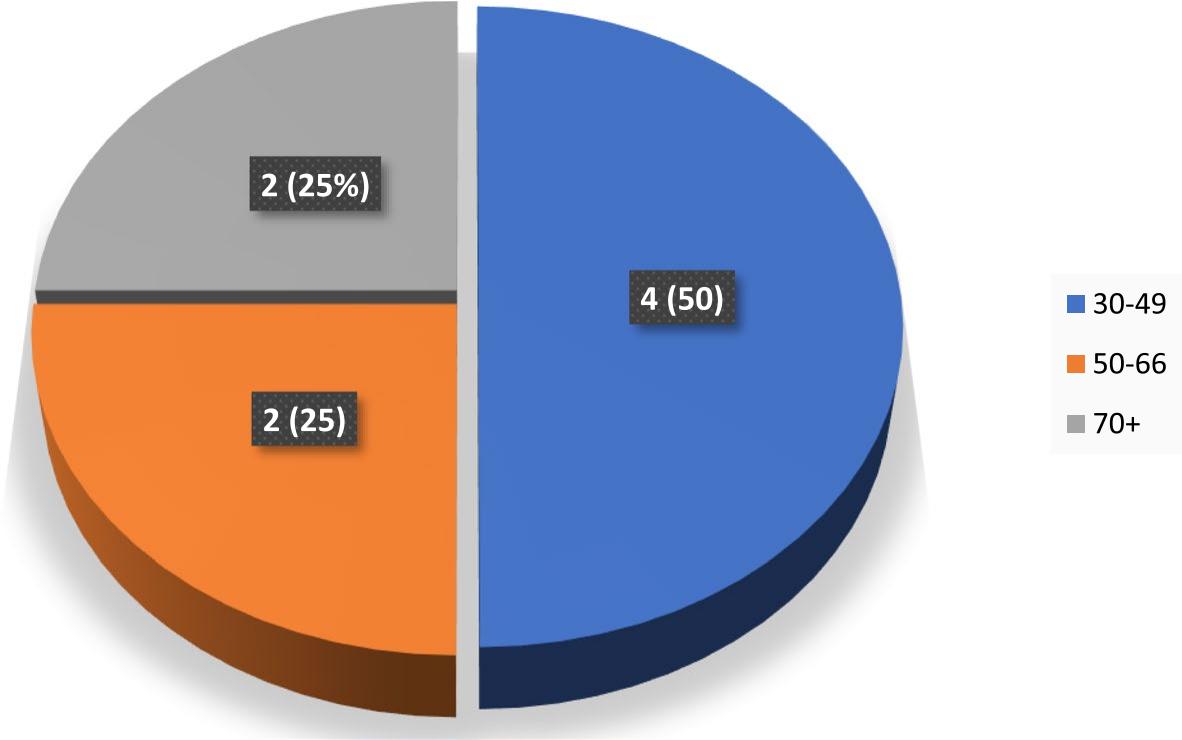
Distribution of LADA patients by age group

The mean BMI of LADA patients was 28.5 (SD, 3.9), and the mean BMI for Type 2 patients was 27.4 (SD, 5.4) (*p-*value = 0.39). Underweight was observed in 7 (4.7%) of Type 2 diabetics and none of LADA patients. The normal weight was observed in 2 (25%) of LADA patients and 43 (29.1%) of Type 2 diabetic patients. The obese and overweight groups in the LADA and Type 2 diabetic patients were observed in 6 (66.2%) and 96 (75%) (Table 1). The study also enabled LADA patients’ stratification by their age groups to show its distribution among the study population (Fig. 1).

Diabetes risk factors were examined, of which 4 (50%) of LADA patients had a history of alcohol intake, which is higher than 55 (37.2%) of Type 2 diabetes (*p =* 0.478). The proportion of history of cigarette smoking was 1 (12.5%) for LADA and 23 (15.5%) for Type 2 diabetes (*p* = 1.00). Family history of first-degree relatives with diabetes among Type 2 diabetes had a higher proportion of 69 (46.6%) and 3 (37.5%) was observed in LADA patients which was not statistically significant (Table 1).

### Comparison of treatment regimen and glycaemic control of LADA to T2D

Among LADA patients who received Biguanide (metformin), 60% were non-adherent (had three and more subsequent visits with borderline Fasting glucose of ≥8 mmol/l). In other LADA patients who received the Biguanide (metformin) in combination with Sulfonylureas, 20% were adherent with good glycaemic control (had three and more subsequent visits with borderline fasting glucose of ≤7.9 mmol/l). For T2D, 5.2% of the patients who received Biguanide (metformin) were adherent with good glycaemic control. The other T2D patients who received the combination of both Biguanide (metformin) and Sulfonylureas had a proportional 32.3% of reasonable glycaemic control. Comparing the patients who used Sulfonylureas only between LADA and T2D, the observation of adherents showed a proportion of 80 and 62.5%, respectively. There was no patient of LADA who was prescribed insulin treatment, while 10% of T2D patients were in insulin treatment with good glycaemic control during the course of diabetes management.

### Comparison of the complication of LADA to T2D patients

**Fig. 2:**
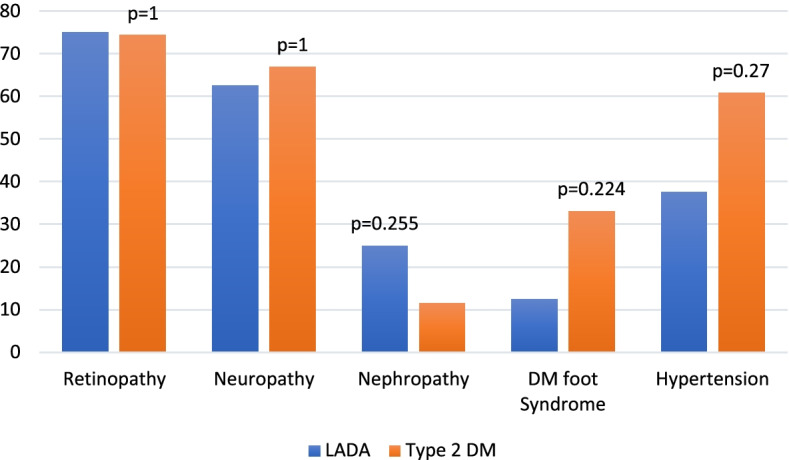
Complications of LADA to Type 2 diabetics’ patients

The complications of patients in their proportions of LADA and Type 2 diabetes indicated retinopathy in 6 (75%) and 110 (74.3%), respectively; neuropathy in 5 (62.5%) and 99 (66.9%), respectively; nephropathy in 2 (25%) and 17 (11.5%), respectively; DM foot syndrome in 1 (12.5%) and 49 (33.1%), respectively and hypertension in 3 (37.5%) and 90 (60.8%), respectively (Fig. 2).

### Comparison of LADA and T2D patients with hypertension and HIV comorbidity

Comparing LADA participants with hypertension and T2D with hypertension, retinopathy was found in 2 (66.7%) and 65 (72.2%), respectively (*p-*value = 0.8333). LADA and Type 2 diabetes proportions with comorbidity of hypertension who had neuropathy were 1 (33.3%) and 60 (66.7%) (*p* = 0.232), respectively. Among the patients of LADA who had hypertension, none of them had nephropathy hence no comparison was made to those of T2D. In the T2D living with hypertension, 10 (11.1%) had a history of nephropathy. With LADA patients living with hypertension, 1 (33.3%) had a complication of Diabetes Mellitus foot syndrome, while in Type 2 diabetes, Diabetes Mellitus foot syndrome was noted in 63 (70%) (*p* = 0.901) (Table 2).

**Table 2:** Summary of LADA and Type 2 patients with hypertension (*n* = 93)

Complication	Total	LADA with Hypertension	Type 2 DM with Hypertension	***P***-value
		*n* = 3	*n* = 90	
	n (%)	n (%)	n (%)	
Retinopathy	67 (72.0)	2 (66.7)	65 (72.2)	0.833
Neuropathy	61 (65.6)	1 (33.3)	60 (66.7)	0.232
Nephropathy	10 (10.8)	0 (0)	10 (11.1)	0.541
DM foot Syndrome	28 (30.1)	1 (33.3)	63 (70.0)	0.901

*LADA* Latent autoimmune diabetes in adults, *DM* Diabetes mellitus

### The complications of LADA to T2D patients with HIV comorbidity

Comparing LADA and T2D with HIV comorbidity, a number of complications were analysed, and the results were as follows. All individuals classified as LADA with HIV comorbidity had retinopathy, while 20 (87%) of T2D with HIV comorbidity had retinopathy (*p* = 0.669). No LADA-HIV comorbid patient had neuropathy, while 22 (95.7%) of T2D patients with HIV had neuropathy (*p* = 0.001). None of the patients with LADA and HIV comorbidity were observed to have nephropathy as a complication, while 3 (13%) of Type 2 diabetic patients with HIV comorbidity had nephropathy, but the differences were not statistically significant (*p-*value = 0.699). No D.M. foot syndrome among patients with LADA and HIV comorbidity, while 2 (8.7%) of T2D with HIV comorbidity had D.M. foot syndrome (*p-*value of 0.758).

## Discussion

In sub-Saharan Africa, scientific evidence on the burden and appropriate management of LADA is very limited; therefore, policies for screening LADA are not in place. Ministries of health in Africa need evidence so they can develop such policies. The study’s primary aim was to establish the prevalence of latent autoimmune diabetes in adults and its associated factors in Dar es Salaam, Tanzania. We, therefore, discuss the established key issues concerning the burden of LADA among T2D patients attending selected health facilities in Dar es Salaam. These concerns include the prevalence of LADA of 5.1% (95% CI 2.5 - 10.0), the absence of screen testing for LADA, and misdiagnosis of LADA that could be associated with related complications.

In the study, LADA has been defined as a subgroup of Type 1 diabetes characterized by slow progressive beta-cell destruction and has been misdiagnosed as T2D. LADA patients had no ketoacidosis at diagnosis, non-insulin required for the first 6 months, aged above 30 years, and had high GAD titre values of ≥200 ng/ml with respect to assay kit used [10, 21, 27]. The proportional estimate (5.1%) of LADA established in this study compares well with the proportional estimate reported in other countries globally and shows some degree of variations from other studies done elsewhere [9, 15, 21, 28, 29]. The study has reflected on the burden of this autoimmune subgroup of diabetes among T2D patients with the increasing proportion that is unnoticeable due to the unavailability of screening programs for LADA prior to and during the initial diagnosis of diabetes in adults patients. Available reports indicate that most of the studies that classify LADA in phenotypically diagnosed T2D have been conducted as cohort studies with more than 1000 subjects [27, 30]. In the U.K., for instance, one prospective diabetes study reported a prevalence of 10%, while Botnia and west Finland reported a prevalence of 9% each [27, 30]. Other studies conducted in North America recruiting < 200 patients have reported prevalence estimates ranging from 3.4 to 16% [31, 32].

The prevalence of LADA obtained among 156 T2D subjects sheds light on the magnitude of LADA in our communities and petition for interventions and larger studies that may lead to policy change in the screening and early diagnosis of T2D patients. The proportional estimate of LADA (5.1%) reported in our study was comparable to another by Lutale et al. [33] in the country, which reported a prevalence of 7.3% of Type 2 diabetes with either GADA or IA2A autoantibodies compared to 43% of Type 1 diabetes with these autoantibodies. The studies done in North America and Europe reported a prevalence of 4.7 and 3.7%, respectively [34]. These proportional estimates compare very well with ours within the 95% CI reported in our study. The different prevalence ranges in different populations might be attributed to ethnicity and genetic variability among different populations, making some individuals more likely to have autoantibodies against islet beta cells of the pancreas [35]. Different studies carried out in the same country (36, 37) may yield a wide range of findings also depending on different methods, study populations, and laboratory autoantibody testing techniques used, thus bringing ambiguities in homogenizing prevalence in the community [9]. To the best of my knowledge, this is the first study in Tanzania to evaluate GAD autoantibody as an independent factor to classify LADA from phenotypically diagnosed T2D. The other study conducted at Muhimbili Nation Hospital diabetic clinic in Dar es Salaam used diabetic-related autoantibody to compare Type 1 and Type 2 diabetic patients [33].

The study suggests that the burden of LADA could be higher in the age group of 30-49 years (50%) than at the later age 50 to 69 and above (Fig. 1). This observation provides clues to the danger of weakening individuals in that age group, which is the most peak productive age in the population. The findings in our study are similar to a previous study by Carlsson et al. [36] that suggested the burden of LADA decreases with increasing age. Another study by Horton et al. [37] suggested that age can influence genetic determination, especially with the genes DRB1/DQB1 in younger LADA subjects in contrast to a specific genotype that occurred after the age of 55.

The distribution of the proportional estimates of the LADA between males and females with Type 2 Diabetes did not show a statistical difference (Table 1). This finding aligns with other studies that reported LADA to not be influenced by individuals’ sex and that the distribution is even unpredictable among LADA and T2D [38]. The findings from our study suggest that LADA patients had relatively higher mean BMI compared to T2D patients, which had no association statistically. The distribution of BMI in LADA patients was observed to be higher in overweight (Table 1). The findings do not exempt a possible relationship of weight as a risk factor for LADA when the sample size increases. This observation, however, is contrary to what was reported by other studies in western countries that LADA individuals have lower BMI compared to T2D despite the difference being not significant statistically [8, 30].

Among LADA patients, a small proportion (20%) under oral hypoglycaemia drugs did not have good glycaemic control. Good glycaemic control was observed in patients under a combination of sulfonylurea and metformin. LADA patients with poor glycaemic control could imply a decreased insulin production and the implication of resistance to oral hypoglycaemic. Of about 37.5% of LADA patients who had poor glycaemic control, none of them were on insulin treatment. Available reports reveal that both LADA and T2D patients tend to have insulin resistance [39–41]. Different approaches for the LADA management reports recommended that sulfonylurea drugs are not regarded as suitable first-line therapy because these drugs may result in early insulin dependence [42].

This study observed histories of risk factors for diabetes which included the history of alcohol drinking in LADA (50%) of patients, the proportion which is higher compared to Type 2 diabetes, cigarette smoking (15%) in T2D which was higher than those found in LADA (12.5%) and family history of diabetes was slightly higher (46.6%) in Type 2 compared to LADA (Table 1). This may signify external factors and not genetics alone may have contributed to the occurrence of LADA. The history of alcohol was higher in the LADA case, which may suggest alcohol drinks play a part in the occurrence of LADA. Cigarette smoking, which has mutagenic tobacco, and a history of diabetes in the family was higher in Type 2 diabetes, suggesting inheritance and genetic influence among diabetic patients. Parallel findings have been reported in other studies in China, Ghana, Sweden, and Australia [15, 24, 38, 43]. The findings from our study were not statistically significant different between LADA and T2D, but this does not mean there is no association; conclusive results may be produced by decreasing the CI by increasing the sample size.

Increased thirst (Polydipsia) and increased urine output (polyuria) were also observed in the study among the LADA and Type 2 patients. The prevalence of these syndromes was slightly the same among the groups. All LADA patients had the frequency of urination at diagnosis, while 130 (87.8%) was observed in T2D (*p* = 0.598. Increased thirst in LADA was 5 (62.5%) and 121 (81.8%) for Type 2 diabetes with *p-*value = 0.182 (Table 1). Although the difference was not statistically significant, this does not eliminate the possible association between LADA and Type 2 diabetes if larger studies of LADA are conducted. However, the findings from our study are contrary to the study by Fourlanos et al. [44], which reported an increase in the prevalence of these syndromes in LADA compared to T2D patients.

In this study, the occurrence of complications did not show significant statistical differences between LADA and T2D patients. The study compared complications among LADA and Type 2 diabetes patients, whereby retinopathy and nephropathy were notably predominant complications in LADA compared to T2D. Neuropathy was found in small proportions in LADA compared to T2D patients (Fig. 2). This is concordant with Adeleye et al. [15] study, whose results reported similar complications. Other complications such as D. M. foot was higher in Type 2 than LADA (Fig. 2). Poor glycaemic control was also reported in previous studies as a risk factor for retinopathy, neuropathy, nephropathy, and D.M. foot complications and detected at a higher proportion in LADA patients’ compared to T2D [15, 38, 45, 46]. The absence of the association of these covariates in this study could be attributed to the sample size obtained in the study for the LADA patients.

The present study sheds light on the comorbidity of LADA with HIV and hypertension. However, all individuals classified as LADA with HIV comorbidity had retinopathy, while a smaller proportion of patients with T2D with HIV comorbidity had retinopathy. No comparison was made on neuropathy, nephropathy, and D.M. foot among LADA and T2D with HIV comorbidity because none of the LADA patients in this stratification had these complications. On the other hand, the study found about 1 out of each tenth of patients with T2D with HIV had nephropathy or D.M. foot syndrome. In comparing LADA and Type 2 diabetes patients with hypertension (Table 2), no evidence increased the frequency of complications among these patients. However, these findings are not conclusive as to which type of diabetes between the LADA and Type 2 diabetes are more prone to complications but justify a need for further research with big sample size. To the best of our knowledge, this stratification is the first of its own and can be explored more in future bigger studies.

### Study limitation and mitigation

We had budget and time limitations, which resulted in a few numbers of cases with LADA that had inadequate power to establish factors associated with LADA conclusively. This could have been attributed to the failure to get a significant factor among all analysed factors that have been reported elsewhere to be significant. However, the findings from this scholarly study could be regarded as preliminary to call for a relatively larger study with larger sample size.

## Conclusion

The study has established the prevalence of LADA among type 2 diabetes patients attending selected health facilities in Dar es Salaam. The study has shown the role of GAD autoantibody in the screening for LADA among T2D. LADA may be associated with serious complications that threaten patients’ quality of life which could have been avoided by proper diagnosis. This study has justified a need for recommending larger and different study designs to develop robust evidence on the association of risk factors and complications associated with the LADA.

## Ethics approval and consent to participate

The ethical clearance was granted by of MUHAS Research Ethics Sub-Committee of the Senate Research and Publication Committee. Ethical Guidelines were implemented during the study. Permission to conduct this study was requested from each hospital. Written informed consent forms were signed by patients before the enrolment of any participant into the study.

## Methods

All methods were carried out in accordance with relevant guidelines and regulations or Declaration of Helsinki.

## Supplementary Information

**Additional file 1.**

**Additional file 2.**

## Data Availability

The datasets used and/or analysed during the current study are available from the corresponding author on reasonable request.
