# Supplementary material for: Prevalence and factors associated with latent autoimmune diabetes in adults (LADA): a cross-sectional study
Source: BMC Endocr Disord. 2022 Jul 8;22:175. doi: 10.1186/s12902-022-01089-1 (PMC9270809; doi:10.1186/s12902-022-01089-1)
Supplement: Supplementary file 2 — Additional file 2. [file 12902_2022_1089_MOESM2_ESM.pdf]

**Objective 1: To determine the proportional of Glutamate Decarboxylase positive among phenotypical Type 2 diabetic patients**

Table 6: Proportion of Glutamate Decarboxylase positive among phenotypical Type 2 diabetic patients, n=156

| **Glutamate Decarboxylase status** | **Frequency** | **Percentage (%)** | **95% CI** |
|---|---|---|---|
| Glutamate Decarboxylase positive | 8 | 5.1 | 2.5 - 10.0 |
| Glutamate Decarboxylase negative | 148 | 94.9 | 90.0 - 97.4 |
| **Total** | **156** | **100** | |

**Glutamate Decarboxylase status among phenotypical Type 2 diabetic patients by patient characteristics, n=156**

| **Clinical histories and characteristics** | **Glutamate Decarboxylase status** | | | |
|---|---|---|---|---|
| | **Total** | **LADA** | **TYPE 2 DM** | **p-value** |
| | | **n= 8** | **n=148** | |
| | | **n (%)** | **n (%)** | |
| **Sex** | | | | |
| Females | 96 (61.5) | 6 (75.0) | 90 (60.8) | 0.711 |
| Males | 60 (38.5) | 2 (25.0) | 58 (39.2) | |
| Age (mean ±SD) | 54.3±11.7 | 55.8±16.6 | 54.2 ±11.5 | 0.135$^{t}$ |
| BMI (mean ±SD) | 27.5±5.3) | 28.5 ±3.9 | 27.4 ±5.4 | 0.309$^{t}$ |
| BMI distribution | | | | |
| Underweight | 7 (4.5) | 0 (0) | 7 (4.7) | 1.00 |
| Normal | 45 (28.9) | 2 (25.0) | 43 (29.1) | |
| Overweight/ Obese | 104 (66.7) | 6 (75.0) | 98 (66.2) | |
| | | | | |
| Age onset of diabetes [median (IQR)] | 52 (41- 62) | 46.5 (38.3-68.5) | 53 (41- 62) | 0.698 |
| Duration of diabetes in years [median (IQR)] | 3 (1 – 4) | 3.5(2.3-5) | 3 (1 - 4) | 0.803 |
| History of alcohol | 59 (37.8) | 4 (50.0) | 55 (37.2) | 0.478 |
| History of cigarettes | 24 (15.4) | 1 (12.5) | 23 (15.5) | 1.00 |
| Family history with diabetes | 72 (46.2) | 3 (37.5) | 69 (46.6) | 0.726 |
| Frequent urination | 138 (88.5) | 8 (100) | 130 (87.8) | 0.598 |
| Ketonuria | 16 (10.3) | 2 (25.0) | 14 (9.5) | 0.192 |

| | | | | |
|---|---|---|---|---|
| Blurred vision | 118 (75.6) | 6 (75.0) | 112 (75.7) | 1.00 |
| Increasing thirst | 126 (80.8) | 5 (62.5) | 121 (81.8) | 0.182 |
| HIV positive (positive only) | 24 (15.4) | 1 (12.5) | 23 (15.5) | 1.00 |
| MTB positive | 12 (7.7) | 0 (0) | 12 (8.1) | 1.00 |
| Oral hypoglycaemia | 139 (89.1) | 8 (100) | 131 (88.5) | 0.60 |
| Fasting blood sugar level at diagnosis (mean, SD) | 21.8 (7.9) | 23.3 (8.1) | 21.8 (7.9) | 0.948$^{t}$ |

*Note: Superscript t indicates p-values obtained from t-test, other P-values obtained from Fisher's Exact test*

**OBJECTIVE 2: T**o compare the glycemic control of LADA to Type 2 diabetics' patients

Table: Treatment choices among adherent LADA and Type 2 diabetics' patients, n=114

| **Treatment choice** | Adherent LADA (GAD POSITIVE) n=5 n(%) | Adherent TYPE 2 (GAD NEGATIVE) n=109 n(%) | P-value |
|---|---|---|---|
| Dietary restriction (yes) | 5 (100) | 109 (100) | - |
| Oral hypoglycaemia (yes) | 5 (100) | 96 (88.1) | 1.00 |
| **Type of oral hypoglycaemia** | | | |
| Biguanide(metformin) | 0 (0) | 2 (5.2) | 0.739 |
| Sulfonylureas | 4 (80.0) | 60 (62.5) | |
| Both metformin and Sulfonylureas | 1 (20.0) | 31 (32.3) | |
| Insulin treatment | 0 (0) | 11 (10.1) | 1.00 |

*P-values obtained from Fisher's Exact*

**OBJECTIVE 3** To compare the complications of LADA to Type 2 diabetics' patients and comparison of complications in stratified groups with comorbidity conditions, including HIV

Table 5: Complications and other associated factors by GAD status, n=156

| | **Total n(%)** | **LADA, n=8 n(%)** | **Type 2 DM, n=148 n(%)** | **P-value** |
|---|---|---|---|---|

| | | | | |
|---|---|---|---|---|
| Retinopathy | 116 (74.4) | 6 (75.0) | 110 (74.3) | 1.000 |
| Neuropathy | 104 (66.7) | 5 (62.5) | 99 (66.9) | 1.000 |
| Nephropathy | 19 (12.2) | 2 (25.0) | 17 (11.5) | 0.255 |
| DM foot Syndrome | 50 (32.1) | 1 (12.5) | 49 (33.1) | 0.224 |
| Hypertension | 93 (59.6) | 3 (37.5) | 90 (60.8) | 0.27 |

*P-values obtained from Fisher's Exact*

Table 5b: comparison of LADA PATIENTS WITH HYPETENSION and TYPE 2 patients with hypertension, n=93

| **Complication** | **Total n(%)** | **LADA, n=3 n(%)** | **Type 2 DM, n=90 n(%)** | **P-value** |
|---|---|---|---|---|
| Retinopathy | 67 (72.0) | 2 (66.7) | 65 (72.2) | 0.833 |
| Neuropathy | 61 (65.6) | 1 (33.3) | 60 (66.7) | 0.232 |
| Nephropathy | 10 (10.8) | 0 (0) | 10 (11.1) | 0.541 |
| DM foot Syndrome | 28 (30.1) | 1 (33.3) | 63 (70.0) | 0.901 |

*P-values obtained from Fisher's Exact*

Table 5c: comparison of LADA PATIENTS with HIV and TYPE 2 patients with HIV, n=24

| **Complication** | **Total n(%)** | **LADA, n=1 n(%)** | **Type 2 DM, n=23 n(%)** | **P-value** |
|---|---|---|---|---|
| Retinopathy | 21 (87.5) | 1 (100) | 20 (87.0) | 0.699 |
| Neuropathy | 22 (91.7) | 0 (0) | 22 (95.7) | 0.001 |
| Nephropathy | 3 (12.5) | 0 (0) | 3 (13.0) | 0.699 |
| DM foot Syndrome | 2 (8.3) | 0 (0) | 2 (8.7) | 0.758 |
| Hypertension | 17 (70.8) | 1 (100) | 16 (69.6) | 0.512 |

*P-values obtained from Fisher's Exact*
